# Hemodynamic and symptomatic response in hypertrophic obstructive cardiomyopathy patients on myosin inhibitor therapy

**DOI:** 10.3389/fcvm.2025.1639855

**Published:** 2026-01-02

**Authors:** Katharina Seuthe, Athanasios Feidakis, Richard Nies, Monique Brüwer, Lenhard Pennig, Kenan Kaya, Henrik ten Freyhaus, Stephan Baldus, Roman Pfister

**Affiliations:** 1 Department III of Internal Medicine, Heart Center, Faculty of Medicine and University Hospital Cologne, University of Cologne, Cologne, Germany; 2 Institute for Diagnostic and Interventional Radiology, Faculty of Medicine and University Hospital Cologne, University of Cologne, Cologne, Germany

**Keywords:** hypertrophic obstructive cardiomyopathy, myosin inhibitor, mavacamten, hemodynamic response, symptomatic response

## Abstract

**Aims:**

To provide real-world data on the symptomatic and hemodynamic response of the myosin inhibitor mavacamten in patients with hypertrophic obstructive cardiomyopathy (HOCM).

**Methods:**

Patients with HOCM up-titrated to their final mavacamten dose were included. The final dose was defined as (i) 15 mg daily (or 5 mg for poor CYP2C19 metabolizers), (ii) a dose achieving a complete hemodynamic response (LVOT gradient <30 mmHg), or (iii) a lower dose limited by adverse effects. Final evaluation was performed 12 weeks after reaching the final dose. Symptomatic response was defined as ≥1 NYHA class improvement, and incomplete hemodynamic response as residual LVOT gradient ≥30 mmHg.

**Results:**

40 patients (56 ± 12 years, 78% male) were included. The LVOT gradient (rest: −25 ± 32 mmHg, *p* < 0.001, Valsalva: −73 ± 56 mmHg, *p* < 0.001) and NT-proBNP levels (−785 ± 1,122 ng/L, *p* < 0.001) significantly decreased during a mean follow up of 184 ± 83 days. 78% had a symptomatic response and 93% were complete hemodynamic responders. Patients with no improvement in NYHA class had a lower e′ lat. (9 ± 3 cm/s vs. 6 ± 2 cm/s, *p* = 0.034) and less often baseline therapy with beta-blockers. Patients with incomplete hemodynamic response had a significantly higher baseline septum thickness (26.3 ± 4.9 mm vs. 19.1 ± 3.7 mm, *p* = 0.007) and higher LV-mass index (205 ± 63 mL/m^2^ vs. 139 ± 31 mL/m^2^, *p* = 0.038). Absolute reduction of LVOT gradients was similar in patients with and without clinical or hemodynamic response.

**Conclusion:**

Clinical and hemodynamic response to mavacamten was high in this real-world cohort and comparable to pivotal trial results. Incomplete response might be related to more severe baseline disease, which needs further study.

## Graphical Abstract

**Figure:**
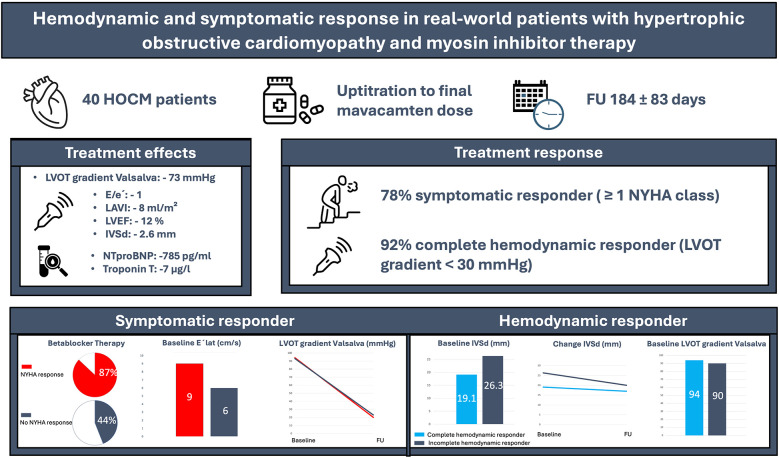
Hemodynamic and symptomatic effects of mavacamten in real-world HOCM patients. Among 40 patients treated over 184 ± 83 days, 78% improved by ≥1 NYHA class and 92% achieved complete hemodynamic response. Therapy led to marked reductions in LVOT gradient, NT-proBNP, troponin, septal thickness, and LAVI. Created using Canva (https://www.canva.com/), licensed under Pro Content License.

## Introduction

Hypertrophic obstructive cardiomyopathy (HOCM) is a genetic cardiac disorder with an estimated prevalence of 1:200–1:500 (1). It is characterized by left ventricular hypertrophy and dynamic left ventricular outflow tract (LVOT) obstruction (2), which can lead to a spectrum of clinical symptoms, including dyspnoea, chest pain, syncope, and an increased risk of sudden cardiac death (1). The management of HOCM traditionally comprised negative inotropic drugs such as beta-blockers, calcium channel blockers (CCB) and disopyramide (3, 4) as well as invasive septal reduction procedures in a more symptomatic disease stage refractory to drug treatment.

Mavacamten is a first-in-class myosin inhibitor addressing the underlying pathophysiology of HOCM through the reduction of myocardial hypercontractility. In pivotal randomized controlled trials (5–8), mavacamten compared to placebo significantly enhanced exercise capacity, reduced LVOT gradient, improved NYHA class and decreased the need for septal reduction therapy (SRT) in patients with symptomatic HOCM. These findings led to the approval of mavacamten in 2023 in Europe and the inclusion into recent guideline recommendations (9, 10).

So far, real-world data of mavacamten are lacking. Therefore, this study aims to investigate patient-specific responses of hemodynamics, clinical status and adverse events in the first 40 patients treated at a tertiary care referral centre.

## Methods

### Study population

This prospective observational study examined patients with HOCM initiating mavacamten treatment at a tertiary care referral centre. The definition of HOCM and indication for initiating mavacamten therapy followed current guideline recommendations (10). As such, treatment was started in symptomatic HOCM patients (NYHA class ≥ II) who had a persistent peak LVOT gradient ≥ 50 mmHg, despite receiving maximum tolerable negative inotropic drug therapy with beta-blockers or CCB with or without disopyramide.

The study included consecutive patients who underwent up-titration of mavacamten and reached their presumed “final” dose. This final dose was defined as one of the following: (i) the maximum approved daily dose of 15 mg (or 5 mg for low CYP2C19 metabolizers), (ii) a dose that achieved a complete hemodynamic response, characterized by a maximum LVOT gradient of <30 mmHg, or (iii) a dose below the maximum of 15 mg due to mavacamten-related side effects even if a complete hemodynamic response was not achieved.

The study was part of a prospective institutional registry of HOCM patients. Written informed consent was obtained from all participants prior to enrolment. The ethics board of the University of Cologne approved the study protocol (23-1481_2).

### Follow-up and up-titration

Patients were initiated on mavacamten therapy and monitored per manufacturer recommendations. Those with unknown or low metabolizer status started at 2.5 mg/day, while non-low metabolizers began at 5 mg/day. CYP2C19 metabolizer status was assessed by genotyping at the initial visit and classified as low, intermediate, rapid, or ultrarapid metabolizers. Dose adjustments were made every 12 weeks, with LVOT gradient and left ventricular function (LVEF) monitored at weeks 4, 8, 12, and subsequently every 12 weeks, including 4 weeks post-adjustment. Each visit included assessments via transthoracic echocardiography (TTE), electrocardiography (ECG), and biomarker analysis. If LVEF dropped <50%, treatment was paused until recovery and restarted at the next lower dose. The “final” follow-up occurred 12 weeks after reaching the final dose without further necessary adjustments.

### Transthoracic echocardiography (TTE)

TTE was conducted in the dedicated echocardiography laboratory with either a GE Vivid E 95 or a GE Vivid S70 (GE Vingmed, Horten, Norway) ultrasound system. Echocardiographic assessment for evaluation of efficacy and safety of mavacamten therapy included of LVOT gradients (at rest, with Valsalva, and during exercise—after 20 squats), LVEF, interventricular septal thickness (IVSd), LV mass index, lateral and septal *E*′ velocities, *E*/*e*′ ratio, and left atrial volume index. LVEF was calculated according to the biplane Simpson method. Parameters reported here were assessed according to current guideline recommendations (11, 12). Image analysis was performed through Tomtec Image-Arena Version 4.6, TomTec, Unterschleissheim, Germany.

### Cardiac magnetic resonance (CMR)

CMR imaging was performed using a 1.5-T magnetic resonance scanner (Philips Ingenia, Philips Healthcare, Best, The Netherlands). Details provided in the supplement. CMR evaluation comprised measurement of native T1, extracellular volume (ECV), and late gadolinium enhancement (LGE).

### Adverse events (AE)

AE related to mavacamten treatment were systematically monitored and documented at every visit. Treatment related AE were predefined as death, new onset of atrial fibrillation (AF), drop in LVEF <50%, episodes of syncope, ventricular tachycardia, atrioventricular block, and symptomatic bradycardia, dizziness and the permanent discontinuation of mavacamten.

### Cardiac biomarkers

Key biomarkers, assessed at every patient visit, were N-terminal pro-B-type natriuretic peptide (NT-proBNP; Elecsys proBNP II assay, Roche Diagnostics, Germany) and troponin T (Elecsys Troponin T hs STAT, Roche Diagnostics, Germany). The cut off values were <0.014 µg/L for troponin and <125 ng/L for NT-proBNP, respectively.

### Statistical analysis

Data analysis was performed using the SPSS statistical software (version 26). Data are shown as absolute values, percentages, medians with interquartile range and means/geometric means with standard deviation, as appropriate. Variables were tested for normal distribution by the Shapiro–Wilk test. For the comparison of continuous variables, the student's *t* test or Mann–Whitney *U*-test was employed, as appropriate. For statistical analysis of longitudinal data, paired t-test or non-parametric related-samples Wilcoxon signed rank test, was applied, as appropriate. The categorical variables were compared using contingency tables and application of the chi-square test or Fisher's exact test. *P*-values <0.05 were considered as statistically significant. Group comparisons were performed between patients with and without symptomatic improvement and between hemodynamic responder subgroups.

## Results

### Study population

**Figure 1:**
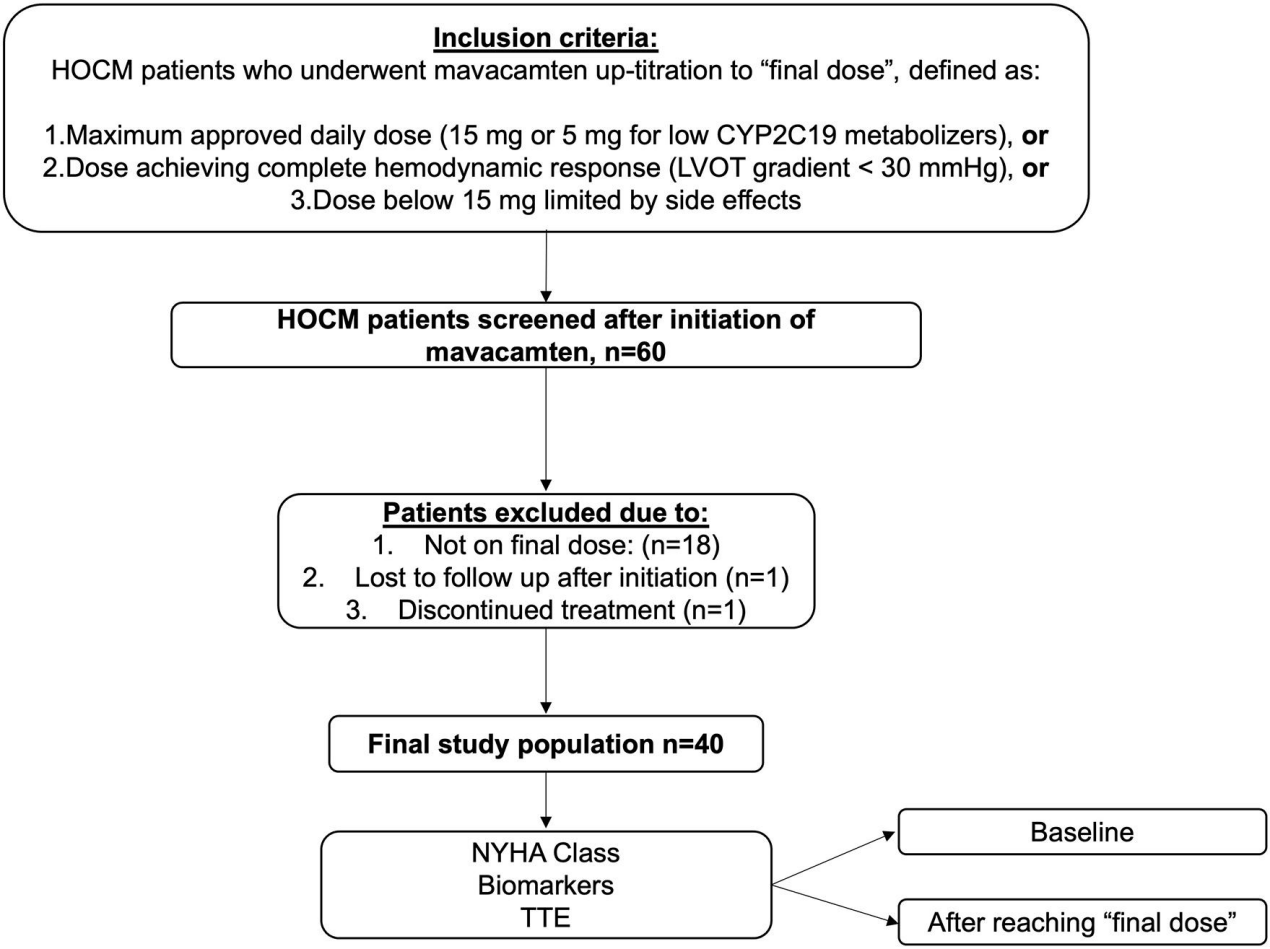
Study protocol and study population. HOCM, hypertrophic obstructive cardiomyopathy; TTE, transthoracic echocardiography; LVOT, left ventricular outflow graft.

60 patients with HOCM initiating mavacamten therapy were initially screened. Of those, 18 were excluded because they had not yet reached the potential final dose, one patient was lost to follow-up, and one discontinued treatment due to worsening dyspnoea, resulting in a final study population of 40 patients (77.5% male, age 56 ± 12 years) included in the analysis (Figure 1). Detailed information on baseline characteristics is shown in Table 1. 14/40 patients (35%) reported dyspnoea NYHA class II, 24/40 (60%) NYHA class III, and 2/40 (5%) NYHA class IV. 7/40 patients (17.5%) had a history of syncope. Baseline therapy consisted of beta-blockers in 32/40 (77.5%) patients, CCB in 6/40 (15%), and disopyramide in 3/40 (7.5%). 5/40 (12.5%) patients had no baseline therapy with negative inotrope drugs due to intolerance with bradycardia or hypotension. Genetic testing was performed in 27/40 (67.5%) patients as per patient decision. In 6/27 (22.2%) patients, a pathogenic or likely pathogenic sarcomeric gene variant reported for hypertrophic cardiomyopathy was found. CYP2C19 metabolizing status was tested in all patients, with detailed distribution provided in Table 1.

**Table 1:** Baseline characteristics.

Variable	All patients (*n* = 40)
Demographics	
Male gender, *n* (%)	31 (77.5)
Age (y), mean (SD)	56.4 (11.9)
Time since diagnosis (years), mean (SD)	3.5 (2.6)
BMI kg/m^2^, mean (SD)	30.2 (5.0)
NYHA functional class, *n* (%)	
I	0 (0)
II	14 (35)
III	24 (60)
IV	2 (5)
Angina Pectoris, *n* (%)	6 (15)
Syncope, *n* (%)	7 (17.5)
Guideline criteria for SRT, *n* (%)	27 (67.5)
Hypertrophic cardiomyopathy genetic testing performed, *n* (%)	27 (67.5)
Pathogenic or likely pathogenic sarcomeric gene variant	6 (22)
CYP2C19 metabolizer status, *n* (%)	
Slow	1 (2.5)
Intermediate	9 (22.5)
Normal	16 (40)
Fast	11 (27.5)
Ultra-fast	3 (7.5)
Comorbidities	
Coronary artery disease, *n* (%)	3 (7.5)
Arterial hypertension, *n* (%)	24 (60)
Atrial fibrillation or flutter, *n* (%)	8 (20)
ICD, *n* (%)	9 (22.5)
Baseline therapy*	
Betablocker, *n* (%)	31 (77.5)
CCB, *n* (%)	6 (15)
Disopyramide, *n* (%)	3 (7.5)
None, *n* (%)	5 (12.5)
Echocardiographic results	
LVEF (%), mean (SD)	71 (7)
IVSd (mm), mean (SD)	19.7 (3.6)
LV mass index (mL/m^2^), mean (SD)	144 (37)
*E*/*e*′, mean (SD)	12 (4)
*E*′lat. (cm/s), mean (SD)	8 (3)
*E*′sept. (cm/s), mean (SD)	6 (2)
LAVI (mL/m^2^), mean (SD)	47 (19)
LVOT gradient (mmHg), mean (SD)	
Rest	37 (31)
Valsalva	93 (55)
Exercise	129 (50)
CMR results (*n* = 30)	
Global T1 relaxation time (ms), mean (SD)	1,081 (55)
Global ECV (%), mean (SD)	29.5 (6.6)
LGE (%), mean (SD)	9 (10)
LVEF (%) mean (SD)	77 (8)
Biomarkers	
NT-proBNP (pg/mL), median (IQR)	516 (952)
Troponin T (µg/L), mean (SD)	18 (13)

* More than one drug per patient possible.

BMI, body mass index, SD, standard deviation; ICD, implantable cardioverter-defibrillator; CCB, calcium channel blockers; LVEF, left ventricular ejection fraction; LAVI, left atrial volume index; LVOT, left ventricular outflow tract; IVSd, interventricular septal thickness.CMR, cardiac magnetic resonance; ECV, extracellular volume; LGE, late gadolinium enhancement; SRT, septal reduction therapy.

### Development over the first 12 weeks

**Figure 2:**
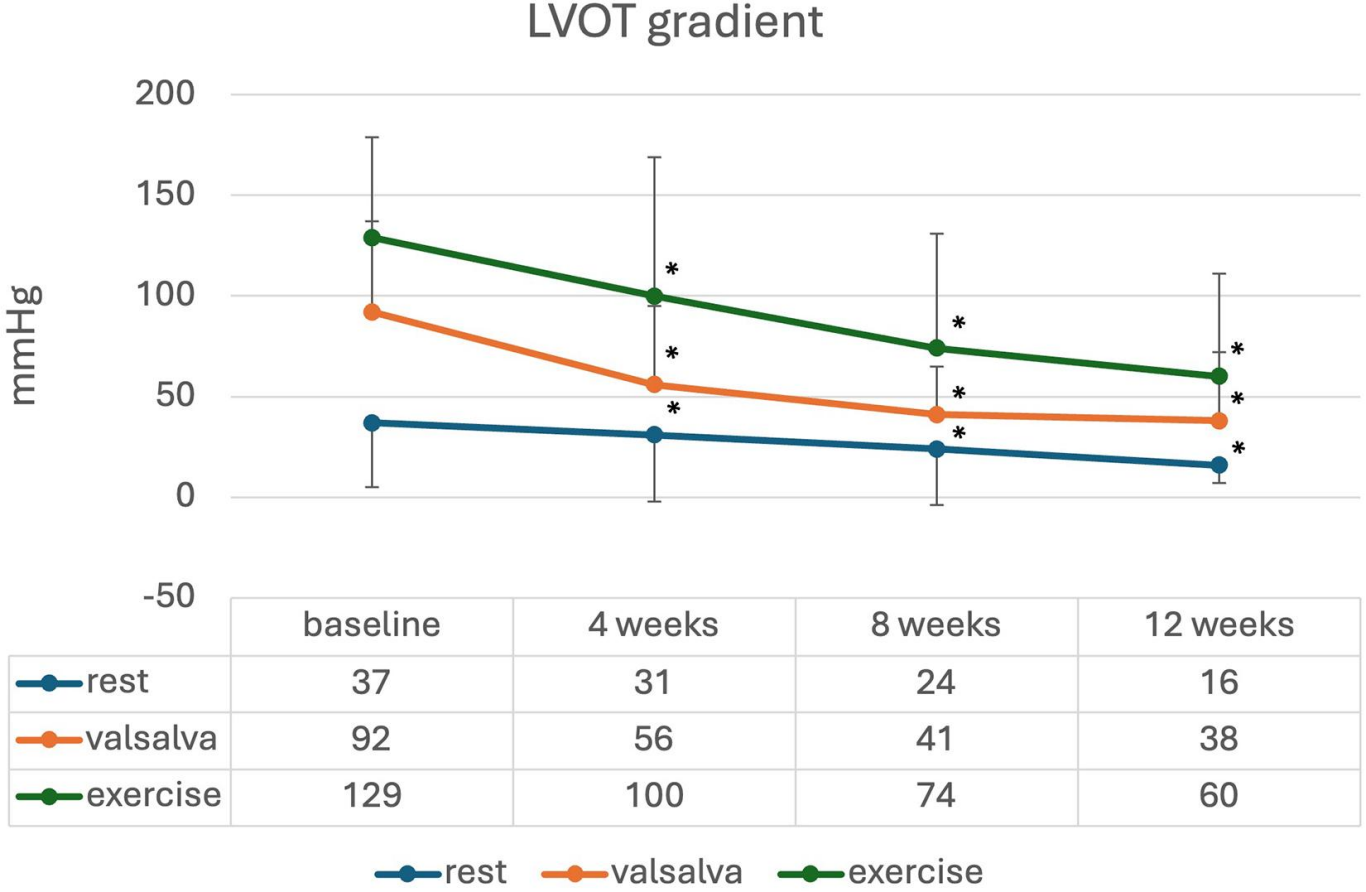
Mean (standard deviation) LVOT gradient during initiation of mavacamten; * significant from baseline *p* < 0.05.

**Figure 3:**
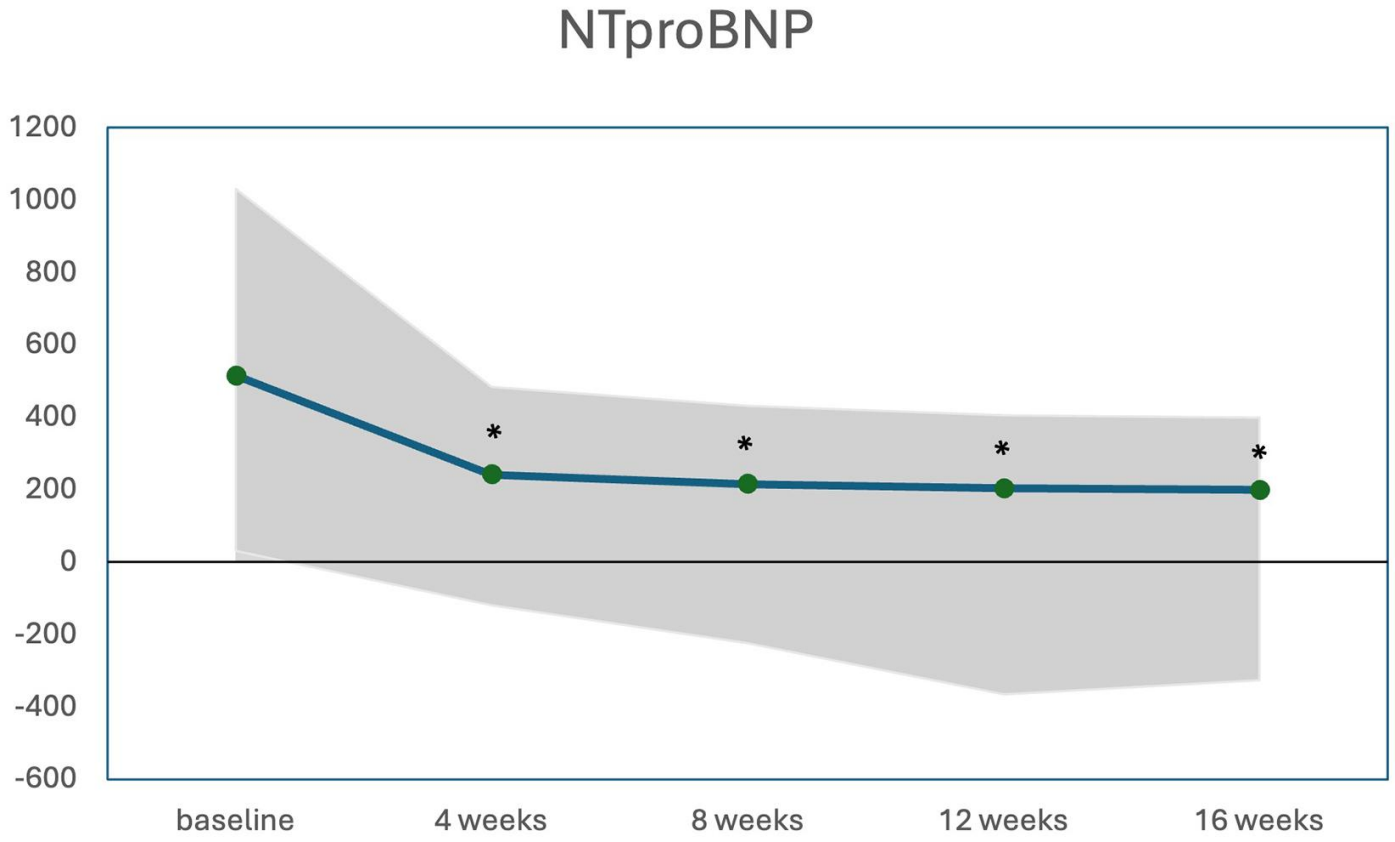
Median (IQR) NTproBNP levels (pg/mL) during initiation of mavacamten; * significant from baseline *p* < 0.05.

The changes in LVOT gradient at rest, during Valsalva, and after exercise, along with NT-proBNP levels over the first weeks of treatment are illustrated in Figures 2, 3. There was a significant reduction in the mean LVOT gradient already after 4 weeks of treatment which further declined at 8, 12 and 16 weeks with all changes being significant compared to baseline. The mean change from baseline to 12 weeks was −24 ± 32 mmHg at rest, −65 ± 82 mmHg at Valsalva and −99 ± 72 mmHg at exercise. Moreover, NT-proBNP levels decreased significantly after 4 weeks (*p* = 0.032) and further decreased until week 16, all values significantly different compared to baseline.

### Effects after reaching the final mavacamten dose

**Figure 4:**
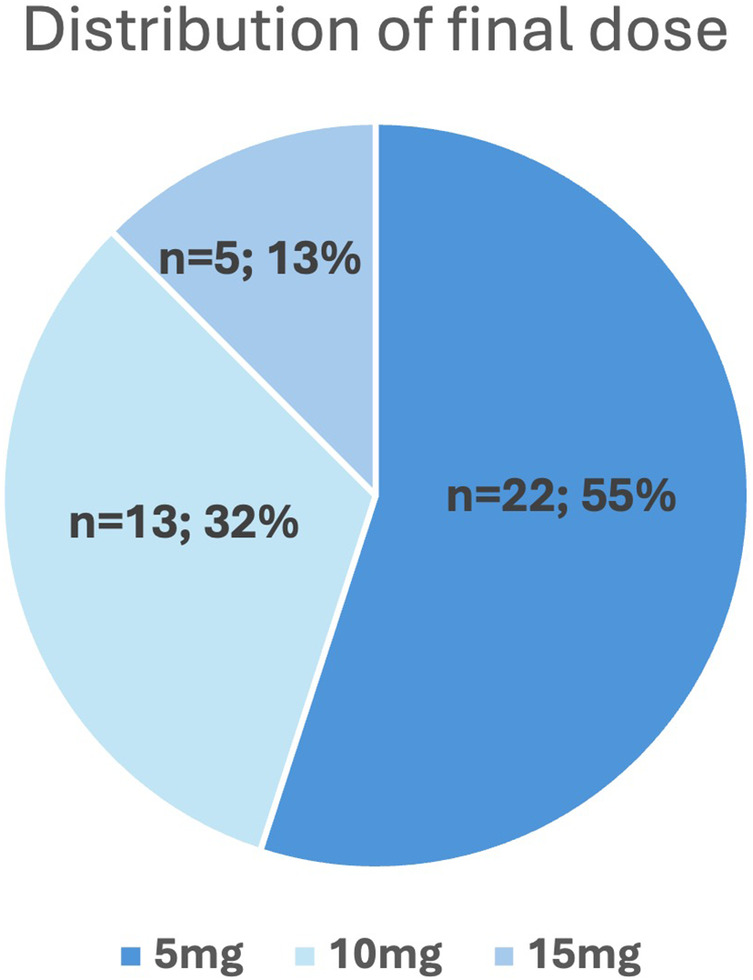
Dose distribution of mavacamten after uptitration to final dose.

The final dose was reached after an average of 92 ± 84 days (median: 77 days, IQR: 116), with the final assessment performed additional 12 weeks later (mean follow up of 184 ± 83 days after starting mavacamten). Notably, more than half (22/40, 55%) of patients achieved complete hemodynamic response with only 5 mg mavacamten, while 18/40 (45%) required a higher dose of 10 or 15 mg (Figure 4). Patients who required a final dose of >5 mg had significantly higher baseline LVOT gradients at rest (26 ± 15 mmHg vs. 50 ± 39 mmHg, *p* = 0.008), under Valsalva (78 ± 43 mmHg vs. 113 ± 61 mmHg, *p* = 0.048) and after exercise (101 ± 39 mmHg vs. 165 ± 45 mmHg, *p* = 0.003). Other baseline characteristics were not different between final dose groups (Supplementary Table S1). Table 2 depicts the changes in echocardiographic parameters and biomarkers from baseline to the final follow up. There was a significant reduction in all LVOT gradients, LVEF, interventricular septal thickness (IVSd), and NT-proBNP and troponin T levels as well as early diastolic mitral inflow velocity to early diastolic annular velocity (*E*/*e*′) and left atrial volume index (LAVI), along with a notable increase in lateral early diastolic annular velocity (e′lat).

**Table 2:** Changes from baseline to final follow up.

Absolute changes from baseline		
Echocardiographic data		*p*
LVEF (%), mean (SD)	−12 (9)	<0.001
IVSd (mm), mean (SD)	−2.4 (2.3)	<0.001
LV mass index (mL/m^2^), mean (SD)	−22 (21)	<0.001
*E*/*e*′, mean (SD)	−1 (4)	0.012
*E*′lat. (cm/s), mean (SD)	+1 (3)	0.005
*E*′sept. (cm/s), mean (SD)	+0.7 (2)	0.028
LAVI (mL/m^2^), mean (SD)	−8 (15)	0.011
LVOT gradient (mmHg), mean (SD)		
Rest	−24 (32)	<0.001
Valsalva	−73 (56)	<0.001
Exercise	−102 (60)	<0.001
Biomarkers		
NT-proBNP (pg/mL), mean (SD)	−785 (1,122)	<0.001
Troponin T (µg/L), mean (SD)	−7 (16)	<0.001

SD, standard deviation; LVEF, left ventricular ejection fraction; LAVI, left atrial volume index; LVOT, left ventricular outflow tract; IVSd, interventricular septal thickness.

### Symptomatic response

**Figure 5:**
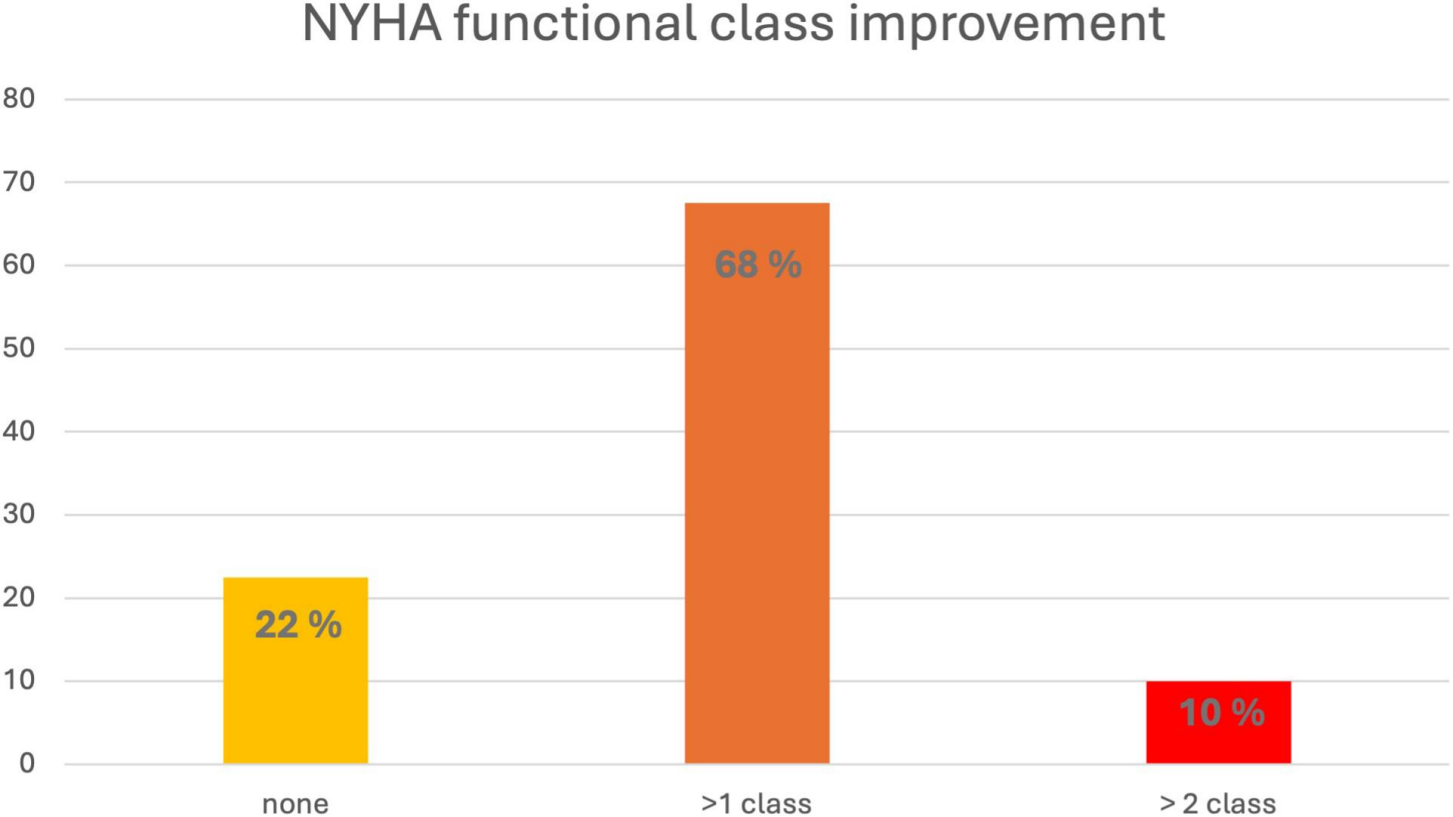
Change in NYHA class (percentage of patients) from baseline to final assessment. NYHA, New York Heart Association.

Figure 5 illustrates the changes in NYHA class between baseline and final follow up. 9/40 (22%) patients showed no change in NYHA class, whereas 31/40 (78%) patients experienced an improvement of at least one NYHA class, with 4/40 (10%) patients improving by 2 NYHA classes. The average time from the start of therapy to a change in NYHA class was 103 ± 99 days. The timing of reported NYHA class improvement correlated with a reduction of the LVOT gradient to <30 mmHg in 10/31 (32%) patients. In 4/31 (13%) cases, NYHA improvement occurred at least 4 weeks after achieving a gradient <30 mmHg, whereas in most patients (15/31, 48%), clinical improvement occurred at least 4 weeks before gradient reduction. In the remaining 2/31 patients with symptomatic response, a LVOT gradient <30 mmHg was not achieved.

At baseline, patients without clinical improvement had significantly lower e′lat., lower NYHA class and lower frequency of beta-blocker use (Table 3). Both groups demonstrated similar treatment effects with respect to changes of LVEF, IVSd, all LVOT gradients and biomarkers with significant reductions by the time of final follow up. The frequency of incomplete hemodynamic response was 6.4% and 11.1% in patients with and without NYHA class improvement (*p* = 0.545).

**Table 3:** Baseline and follow up of patients with improvement of at least 1 NYHA class vs. no improvement.

	Improved NYHA *n* = 31			No NYHA change *n* = 9				
	Baseline	Change from baseline	*p*-value for change from baseline	Baseline	Change from baseline	*p*-value for change from baseline	*p*-value for comparison of baseline values across groups	*p*-value for comparison of changes from baselineacross groups
Demographics								
Male gender, *n* (%)	24 (77.4)			7 (77.8)			0.680	
Age (y), mean (SD)	56.0 (12.8)			57.8 (8.1)			0.704	
BMI (kg/m^2^), mean (SD)	29.9 (5.0)			29.7 (5.6)			0.905	
BMI > 30	15 (48.4)			2 (22.2)			0.256	
NYHA functional class, *n* (%)								
I/II	8 (28.8)			6 (66.7)			0.044	
III/IV	23 (74.2)			3 (33.3)				
Baseline therapy								
Betablocker, *n* (%)	27 (87)			4 (44.4)			0.016	
CCB, *n* (%)	5 (16.1)			1 (11.1)			0.143	
Disopyramide, *n* (%)	2 (6.5)			1 (11.1)			0.432	
None, *n* (%)	2 (6.5)			3 (33.3)			0.065	
Echocardiographic data								
LVEF (%), mean (SD)	71 (8)	−13 (8)	<0.001	74 (8)	−11 (10)	0.009	0.216	0.734
LV mass index (mL/m^2^), mean (SD)	146 (41)	−23 (23)	<0.001	136 (24)	−19 (15)	0.003	0.522	0.610
IVSd (mm), mean (SD)	19.8 (4.1)	−2.4 (2.5)	<0.001	19.2 (1.5)	−2.7 (1.3)	<0.001	0.827	0.544
*E*/*e*′, mean (SD)	12 (4)	−2 (3)	0.035	14 (4)	−1 (6)	0.161	0.468	0.813
*E*′lat. (cm/s), mean (SD)	9 (3)	+1 (3)	0.048	6 (2)	−2 (3)	0.089	0.034	0.371
*E*′sept. (cm/s), mean (SD)	6 (2)	+1 (2)	0.089	6 (1)	+1 (2)	0.107	0.468	0.702
LAVI (mL/m^2^), mean (SD)	45 (17)	−8 (1)	0.027	53 (23)	−7 (13)	0.214	0.962	0.557
LVOT gradient (mmHg), mean (SD)								
Rest	40 (34)	−27 (35)	<0.001	30 (18)	−17 (17)	0.016	0.429	0.463
Valsalva	94 (51)	−76 (54)	<0.001	93 (67)	−70 (65)	0.009	0.962	0.479
Exercise	129 (43)	−114 (47)	<0.001	105 (66)	−83 (79)	0.630	0.324	0.154
CMR imaging data (*n* = 30)								
Global T1 relaxation time (ms), mean	1,075 (60)			1,099 (33)			0.334	
Global ECV (%), mean (SD)	30.6 (7.1)			26.3 (3.3)			0.136	
LGE (%), mean (SD)	9 (12)			9 (3)			0.719	
LV mass index (mL/m^2^), mean (SD)	121 (32)			115 (23)			0.598	
Biomarkers								
NT-proBNP (pg/mL), mean (SD)	916 (991)	−692 (891)	<0.001	1,408 (1,736)	−1,105 (1,736)	0.045	0.616	0.809
Troponin T (µg/L), mean (SD)	21 (14)	−10 (11)	<0.001	15 (13)	−4 (8)	0.241	0.308	0.075
Incomplete hemodynamic responder	2 (6.4)			1 (11.1)				0.545

SD, standard deviation; CCB, calcium channel blockers; LVEF, left ventricular ejection fraction; LAVI, left atrial volume index; LVOT, left ventricular outflow tract; IVSd, interventricular septal thickness.

### Hemodynamic response

In 37/40 (93%) patients, a complete hemodynamic response with a maximum LVOT gradient <30 mmHg was achieved. The average time from baseline to a reduction in the gradient <30 mmHg was 130 ± 76 days. The time from initiating the final dose to observing a reduction in the gradient <30 mmHg was 50 ± 28 days.

In 3/40 (7.5%) patients the maximum LVOT gradient remained ≥30 mmHg, in 3/3 ≥ 50 mmHg, despite receiving the maximum mavacamten dose of 15 mg/day for 12 weeks. These patients were classified as incomplete hemodynamic responders. Notably, 2/3 patients showed clinical improvement of one NYHA class, while one patient did not show a NYHA class change and was still eligible for SRT at the final follow up.

11/40 (27.5%) patients had complete response, defined as achieving NYHA class I and LVOT gradient <30 mmHg at the final follow up.

**Figure 6:**
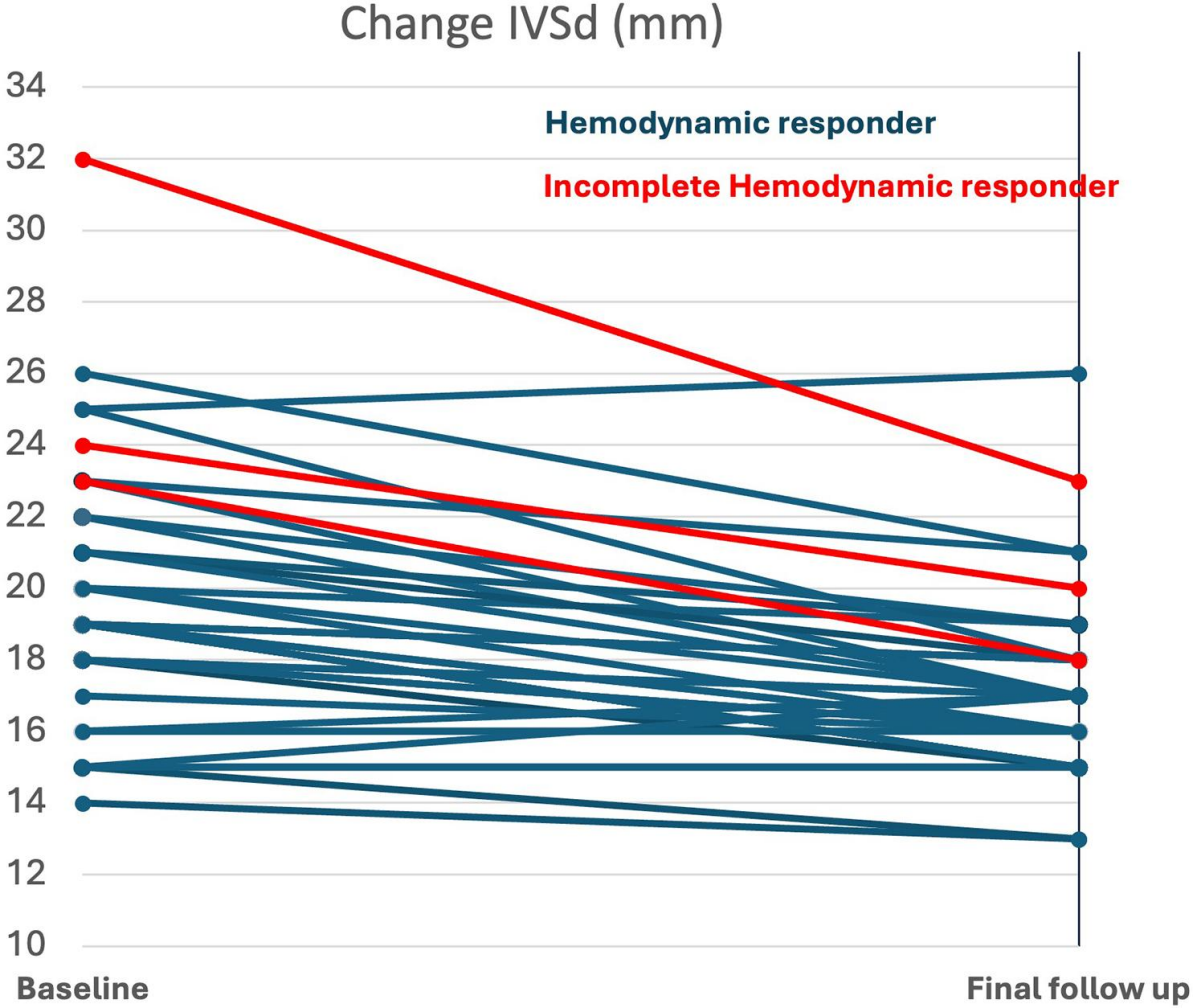
Baseline and change of IVSd in complete and incomplete hemodynamic responders (*p* = 0.007 and *p* = 0.012 for comparison across responder groups); IVSd, interventricular septal thickness.

The 3 patients with incomplete hemodynamic response had significantly higher baseline IVSd (26.3 ± 4.9 mm vs. 19.1 ± 3.7 mm, *p* = 0.007; Figure 6), higher LV-mass index (205 ± 63 mL/m^2^, vs. 139 ± 31 mL/m^2^, *p* = 0.038) and a trend of higher troponin (19 ± 13 µg/L, vs. 35 ± 9 µg/L, *p* = 0.07) at baseline (Supplementary Table S2). However, there was no difference in LVOT gradients at baseline between the two groups. The echocardiographic treatment effect was similar for both groups; there was no significant difference in reduction of LVEF or LVOT gradients. In contrast, the difference in reduction of IVSd and LV-mass index was in favour of the incomplete responders (−6.0 ± 2.6 mm vs. −1.1 ± 2.6 mm, *p* = 0.012), (Figure 5, Supplementary Table S2). All patients with incomplete hemodynamic response had baseline therapy with either beta-blocker or CCB.

### Adverse events

One patient (2.5%) experienced a syncope episode after exercise within the first four weeks of treatment. Three patients (7.5%) reported dizziness during the initial four weeks, either upon therapy initiation or during up-titration to the next higher dose. In all cases, these symptoms resolved during treatment without any dose adjustments. Additionally, there was one case of new-onset atrial fibrillation, successfully treated with cardioversion, and one case of non-sustained ventricular tachycardia (nsVT), which subsequently required ICD implantation; no dose adjustments were made in either case. Notably, one patient (2.5%) experienced a decline in LVEF to below 50% while on the highest mavacamten dose of 15 mg, necessitating a temporary four-week treatment interruption. After the pause, LVEF normalized, and therapy was resumed at the next lower dose, allowing the patient to continue treatment with a full hemodynamic response.

### Discontinuations

One of the 60 screened patients discontinued mavacamten because of worsening dyspnea and was excluded from the study. Among the 40 included patients, no permanent discontinuations occurred during the study period. Temporary dose interruptions were implemented only in the one patient with a transient reduction in LVEF, after which therapy was safely resumed at the next lower dose.

## Discussion

Our study on a real-world cohort of patients treated with mavacamten has the following findings:

(i) Initiation of mavacamten treatment led to symptomatic benefit in 78% and a complete hemodynamic response in 93% of HOCM patients with an excellent safety profile.
(ii) The necessity of an extended up-titration due to higher final doses (>5 mg) correlates with higher LVOT gradients at baseline.
(iii) Symptomatic non-responders and patients with incomplete hemodynamic response showed virtually the same absolute effect on echocardiographic parameters and biomarkers as responders, in particular the same reduction in LVOT gradient.
(iv) Higher baseline LV mass and diastolic dysfunction were found in patients with impaired response, which requires further analysis.

### Study population

Our cohort was broadly representative of contemporary HOCM patients, as described in approval trials (6, 8) and recently published real-world studies (13–15). Compared to trial populations, our patients demonstrated higher baseline gradients during both Valsalva and exercise, even exceeding those observed in the VALOR-HCM cohort, which included high-risk patients eligible for SRT. With two thirds of our cohort meeting criteria for SRT eligibility, our population represents a sicker and more severely symptomatic cohort than EXPLORER-HCM and to other real-world studies (14).

### Treatment effect of mavacamten

We observed rapid and sustained improvements in both hemodynamics and biomarkers, with some effects even more pronounced than in the EXPLORER or VALOR trials (6, 16), particularly regarding the absolute reduction in LVOT gradients. In our cohort, the mean exercise gradient decreased by 102 mmHg, compared with an 80 mmHg reduction reported in other real-world data (14) and only a 47 mmHg reduction in EXPLORER. These pronounced effects are likely attributable to the higher baseline gradients observed in both our cohort and other real-world populations.

Our study showed significant improvements in key parameters of diastolic function, including E/e′, LAVI, e′lat., and e′med. These results complement recent data of an extended follow-up analysis of the VALOR trial (17). This is of particular relevance, since preliminary data suggest that improvements in diastolic function might be more relevant for the symptomatic benefit of patients than reductions in LVOT gradients (16).

A higher percentage of patients (78%) showed a functional improvement compared to those in the VALOR-HCM and EXPLORER-HCM cohorts. This might be due to differences in patient characteristics, nonetheless our data underscore the high efficacy of mavacamten in improving symptoms in a real-world setting. Notably, this is comparable to other real-world data, which reported improvement by ≥1 NYHA class in 72% of patients after ≥6 months of mavacamten therapy (14). The symptomatic benefit temporally correlated with achieving the final dose, which is approximately 9 weeks for the lower mavacamten dose and about 18 weeks for the higher dose. This information could be helpful for patients and physicians, as it demonstrates the expected timeline of improvement.

We observed a 93% rate of complete hemodynamic response with reducing the maximum LVOT gradient to <30 mmHg. This is higher than the results in the EXPLORER-HCM or VALOR-HCM studies but more comparable to those in MAVA-LTE (18) and other longer-term real-world data (14), suggesting a prolonged time to the full treatment effect of mavacamten. Of note, follow-up time in our study resulted from the requirement of a maximum hemodynamic effect. The longest time period to achieve maximum LVOT gradient reduction in our cohort was 370 days, a timeframe not captured in initial trials or most existing real-world data.

### Dose finding

We would like to highlight that the majority of patients (55%) achieved a hemodynamic response at a final mavacamten dose of 5 mg, whereas 35% required 10 mg and only 15% the maximum dose of 15 mg. This differs from existing trials and other real world cohorts, where the 4 different doses (2.5, 5, 10 and 15 mg) were more evenly distributed (14, 18). Notably, that study also demonstrated minimal need for long-term dose adjustments after 48 months (18). None of our patients was finally on the 2.5 mg dose of mavacamten, despite a similar proportion of low metabolizers in our cohort (2.5%) compared to the Explorer cohort (6%). This difference is attributable to a distinct dose finding strategy in our institution. Our patients were directly initiated on 5 mg or were typically increased to 5 mg after 4 weeks, depending on whether the results of metabolizing status were known already at initiation or early after initiation. Additionally, our patients were not systematically down-titrated after achieving complete hemodynamic response which is recommended by the manufacturer in an attempt to apply the lowest necessary mavacamten dose. After initial experiences with recurrence of LVOT gradients after tentatively dose decrease we only down-titrated the mavacamten dose with complete hemodynamic response in the case of side effects.

We observed that a higher final dose is associated with higher baseline LVOT gradient. This insight may assist physicians in planning for longer uptitration periods.

### Symptomatic response

22% of our patients showed no improvement in NYHA class despite maximum tolerable mavacamten dose or complete hemodynamic response. So far, data on clinical non-response of mavacamten therapy are lacking. Both groups, with and without NYHA class improvement, exhibited similar treatment effects in terms of LVOT gradient, diastolic function, and biomarkers. This emphasizes that the height of LVOT gradient on its own does not explain the severity of symptoms in HOCM patients. Importantly with respect to safety, a more pronounced decrease in LVEF did also not explain differences in clinical response. The only other parameter different between responders and non-responders was baseline e′lat. Whether advanced diastolic dysfunction impacts clinical response to mavacamten therapy might need further study in larger populations. Nonetheless, our findings suggest that a lack of NYHA response might not indicate treatment failure of mavacamten therapy with respect to reduction of LVOT gradients.

Of note, the group of clinical non-responders was less often treated with betablocker (*p* = 0.016), although there was no significant difference in percentage of patients without any negative inotropic baseline therapy (*p* = 0.065). Recent data show no difference in functional effects by beta-blocker use suggesting that our findings might be rather chance than a causal relation (17).

### Hemodynamic response

Patients without complete hemodynamic response demonstrated a similar absolute treatment effect in LVOT gradient reduction as those who were complete hemodynamic responders. Comparison of baseline characteristics revealed that incomplete hemodynamic responder had significantly higher IVSd and LV mass index, along with a trend toward elevated troponin levels, indicating either more advanced or more severe disease stage.

This observation raises the question of whether earlier treatment initiation is indicated and or whether doses higher than 15 mg might be necessary to achieve optimal hemodynamic results in selected patients. Results from Phase 2 trials (PIONEER- HCM) (19) suggested higher doses (15–20 mg) had more effect on lowering LVOT gradient but required closer cardiac and pharmacological drug monitoring.

### Adverse events

One patient (2.5%) developed a LVEF drop <50%, and this was under the highest mavacamten dose. The course of further treatment remained uneventful on the lower dose of 10 mg. Overall, our real-world data on adverse events align with those observed in the controlled clinical trial settings (6, 8, 20), supporting the safety profile of mavacamten provided that recommended monitoring is followed.

## Limitations

The present study is a single—centre, uncontrolled study of moderate size with inherent limitations. This single-center, uncontrolled study of moderate size has inherent limitations. The absence of a control group restricts causal inference, particularly for patient-reported outcomes such as NYHA class. The relatively short mean follow-up period precludes assessment of long-term efficacy and safety. Given the exploratory nature of the study, no adjustments for multiple testing were applied, and findings should be interpreted as hypothesis-generating. Multivariate analyses to account for potential confounders were not feasible due to the small sample size, especially in non-responder subgroups. The small size of the incomplete hemodynamic responder subgroup (*n* = 3) limits the statistical reliability of comparative analyses. Additionally, as the cohort was predominantly male (78%), the findings may not be generalizable to the broader HOCM population, particularly regarding sex-specific response patterns.

Nonetheless, our findings provide first real-world experience which supports trial results and might be of interest both for treating physicians and patients. Larger samples are necessary to further elucidate factors associated with incomplete clinical and hemodynamic response. A more sophisticated measure of symptomatic burden with specific questionnaires (21) might also help better understand deficits in clinical response.

## Conclusions

The efficacy of the first myosin inhibitor mavacamten in reducing LVOT gradient and improving NYHA class in our real-world cohort was at least as high as in previous approval trials. In about half of the patients, particularly those with lower baseline LVOT gradients, a final mavacamten dose of 5 mg achieved complete hemodynamic response and symptomatic benefit within the first 4 weeks. Incomplete clinical and hemodynamic response might be related to more severe baseline disease, and further study is warranted exploring the potential role of additional target parameters of mavacamten therapy such as diastolic dysfunction and myocardial mass.

## Data Availability

The original contributions presented in the study are included in the article/Supplementary Material, further inquiries can be directed to the corresponding author.
