# Supplementary material for: Hemodynamic and symptomatic response in hypertrophic obstructive cardiomyopathy patients on myosin inhibitor therapy
Source: Front Cardiovasc Med. 2026 Jan 2;12:1639855. doi: 10.3389/fcvm.2025.1639855 (PMC12808423; doi:10.3389/fcvm.2025.1639855)
Supplement: Supplementary file 1 [file Datasheet1.docx]

Supplementary data

|  | **Final dose 5mg**  **N= 22** | **Final dose >5mg**  **N=18** | **p-value** |
| --- | --- | --- | --- |
| ***Demographics*** |  |  |  |
| Male gender, n (%) | 17 (77.3) | 14 (77.8) | 0.636 |
| Age (y), mean (SD) | 55.7 (11.4) | 57.2 (12.8) | 0.687 |
| BMI kg/m², mean (SD) | 29.3 (4.9) | 30.5 (5.4) | 0.488 |
| NYHA functional class, n (%) I  II  III  IV | 0  9 (40.9)  13 (59.1)  0 | 0  5 (27.8)  11 (61.1)  2 (11.1) | 0.230 |
| ***Baseline therapy***  Betablocker, n (%)  CCB, n (%)  Disopyramide, n (%)  None, n (%) | 16 (72.3)  5 (22.7)  1 (4.5)  3 (13.6) | 15 (83.3)  1 (5.6)  2 (11.1)  2 (11.1) | 0.341  0.143  0.432  0.549 |
| ***Echocardiographic data*** |  |  |  |
| LVEF (%), mean (SD) | 70 (7) | 72 (73) | 0.120 |
| LV mass index (mL/m²), mean (SD) | 136 (34) | 149 (49) | 0.331 |
| IVSd (mm), mean (SD) | 19.3 (3.5) | 19.8 (4.3) | 0.689 |
| E/e´, mean (SD) | 13 (4) | 12 (5) | 0.844 |
| E´lat (cm/s), mean (SD) | 8 (2) | 8 (3) | 0.409 |
| E´sept (cm/s), mean (SD) | 6 (2) | 6 (2) | 0.910 |
| LAVI (ml/m²), mean (SD) | 46 (15) | 47 (22) | 0.517 |
| LVOT gradient (mmHg), mean (SD)   - Rest - Valsalva - Exercise | 26 (15)  78 (43)  101 (39) | 50 (39)  113 (61)  165 (45) | 0.008  0.048  0.003 |
| **Supplementary Table 1:** **Baseline data from patients with final dose 5 vs > 5mg.** SD= standard deviation; CCB= calcium channel blockers; LVEF=left ventricular ejection fraction; LAVI= left atrial volume index; IVSd= interventricular septal thickness. | | | |

|  | **Complete hemodynamic**  **responder**  **N=37** | |  | **Incomplete hemodynamic responder**  **N=3** | |  | **p-value for comparison of baseline values across groups** | **p-value for comparison of changes from baseline**  **across groups** |
| --- | --- | --- | --- | --- | --- | --- | --- | --- |
|  | **Baseline** | **Change from baseline** | **p-value change from baseline** | **Baseline** | **Change from baseline** | **p-value change from baseline** |  |  |
| ***Demographics*** |  |  |  |  |  |  |  |  |
| Male gender, n (%) | 28 (75.7) |  |  | 3 (100) |  |  | 0.455 |  |
| Age (y), mean (SD) | 56.9 (11.8) |  |  | 50.3 (12.6) |  |  | 0.363 |  |
| BMI kg/m², mean (SD) | 29.9 (4.8) |  |  | 29.5 (9.8) |  |  | 0.910 |  |
| NYHA functional class, n (%) |  |  |  |  |  |  |  |  |
| I  II  III  IV | 0  14 (37.8)  21 (56.8)  2 (5.4) |  |  | 0  0  3 (100)  0 |  |  | 0.339 |  |
| ***Baseline therapy***  Betablocker, n (%)  CCB, n (%)  Disopyramide, n (%)  None, n (%) | 29 (78.4)  5 (13.5)  3 (8.1)  5 (13.5) |  |  | 2 (66.7)  1 (33.3)  0  0 |  |  | 0.545  0.394  0.786  1.00 |  |
| ***Echocardiographic data*** |  |  |  |  |  |  |  |  |
| LVEF (%), mean (SD) | 70 (7) | -12 (8) | <0.001 | 79 (9) | -14 (12) | 0.104 | 0.103 | 0.605 |
| IVSd (mm), mean (SD) | 19.1 (33.0) | -2.2 (2.0) | < 0.001 | 26.3 (4.9) | -6.0 (2.6) | 0.109 | 0.007 | 0.012 |
| LV mass index (mL/m²), mean (SD) | 139 (31) | -21 (19) | < 0.001 | 205 (63) | -42 (36) | 0.109 | 0.038 | 0.223 |
| E/e´, mean (SD) | 12 (4) |  |  | 16 (4) |  |  | 0.196 |  |
| E´lat (cm/s), mean (SD) | 8 (3) |  |  | 6 (1) |  |  | 0.136 |  |
| E´sept (cm/s), mean (SD) | 6 (1) |  |  | 5 (1) |  |  | 0.310 |  |
| LAVI (ml/m²), mean (SD) | 48 (19) |  |  | 40 (7) |  |  | 0.941 |  |
| LVOT gradient (mmHg), mean (SD) |  |  |  |  |  |  |  |  |
| -Rest  -Valsalva | 37 (32)  94 (56) | -25 (33)  -78 (56) | < 0.001  < 0.001 | 31 (19)  90 (43) | -17 (11)  -43.3 (36) | 0.109  0.109 | 0.941  0.895 | 0.085  0.347 |
| ***Biomarkers*** |  |  |  |  |  |  |  |  |
| NT-proBNP (pg/mL), median (IQR) | 515 (953) |  |  | 605 |  |  | 0.771 |  |
| Troponin T (µg/l), mean (SD) | 19 (13) |  |  | 35 (9) |  |  | 0.070 |  |
| Maximal possible mavacamten dose | 2 (5.4%) |  |  | 3 (100%) |  |  |  |  |
| ***Metaboliser status*** |  |  |  |  |  |  |  |  |
| Slow  Intermediate  Normal  Fast  Super fast | 0  9  15  10  3 |  |  | 1  0  1  1  0 |  |  |  |  |
| **Supplementary Table 2: Baseline data and data at final follow up from patients with complete vs incomplete hemodynamic responder.** SD= standard deviation; CCB= calcium channel blockers; LVEF=left ventricular ejection fraction; LAVi= left atrial volume index; LVOT= left ventricular outflow tract; CMR= cardiac magnetic resonance; ECV= extracellular volume; LGE= late gadolinium enhancement; IVSd= interventricular septal thickness. | | | | | | | | |

*Cardiac magnetic resonance (CMR)*

CMR imaging was performed using a 1.5-T magnetic resonance scanner (Philips Ingenia, Philips Healthcare, Best, The Netherlands), equipped with a 28-channel coil for cardiac imaging. Sequences were acquired in end-diastole during an end-expiratory breath-hold. The protocol comprised 2D balanced steady-state free precession cine sequences in standard orientations (short axis [SA], 4-chamber [4Ch], 2-chamber [2Ch], and 3-chamber [3Ch]). Prior to admission of gadolinium contrast, T1-mapping (using a 5(3)3 modified look-locker inversion recovery (MOLLI) sequence) was acquired in SA (3 slices; apical, mid-ventricular, and basal). Afterwards, a single bolus of Gadobutrol (Gadovist, Bayer HealthCare Pharmaceuticals, Berlin, Germany; 0.2 mmol/kg) was automatically injected into an antecubital vein (flowrate of 2 mL/s). 10 min after injection of gadolinium contrast, T1-mapping (using a 4(1)3(1)2 MOLLI sequence) was acquired in SA (3 slices). T1-weighted inversion-recovery fast spoiled gradient-echo sequences in standard orientations (SA, 4Ch, 2Ch, and 3Ch) were used for late gadolinium enhancement (LGE) imaging.

Using commercially available software (IntelliSpace Portal version 10.1; Philips Healthcare, Best, The Netherlands), two radiologists with 3 (KK) and 7 (LP) years of experience in CMR analyzed the data and performed the measurements in consensus. Readers were blinded to clinical data. All volumes and masses were indexed to the body surface area at the day of the examination using the Mosteller method (1). Papillary muscles were included in left ventricular mass (2). Myocardial T1-relaxation times and ECV fraction values (using pre- and post-contrast T1 values) were calculated employing a segmental approach (2). After manual delineation of a region of interest in remote myocardium (defined as a segment with neither enhancement nor wall motion disorder), LGE volume was semi-automatically quantified using the 6-standard deviation (SD) technique in SA (2).

1. Mosteller RD. Simplified calculation of body-surface area. Vol. 317, The New England journal of medicine. United States; 1987. p. 1098.

2. Schulz-Menger J, Bluemke DA, Bremerich J, Flamm SD, Fogel MA, Friedrich MG, et al. Standardized image interpretation and post-processing in cardiovascular magnetic  resonance - 2020 update : Society for Cardiovascular Magnetic Resonance (SCMR): Board of Trustees Task Force on Standardized Post-Processing. J Cardiovasc Magn Reson. 2020 Mar;22(1):19.
